# Serpiginous Choroiditis After COVID-19 Infection

**DOI:** 10.1177/24741264241297936

**Published:** 2024-11-14

**Authors:** Sorayya Seddigh, Ashlyn Pinto, Amr M. Zaki, R. Rishi Gupta

**Affiliations:** 1 Department of Ophthalmology and Visual Sciences, Dalhousie University, Halifax, NS, Canada

**Keywords:** serpiginous choroiditis, uveitis, posterior uveitis, COVID-19, SARS-CoV-2, retinal/choroidal inflammation

## Abstract

**Purpose:** To present the first case of macular serpiginous choroiditis after COVID-19 infection. **Methods:** A single case was analyzed. **Results:** A 28-year-old previously healthy man presented with severe unilateral vision loss in the left eye. A fundus examination showed severe atrophic pigmentary changes that corresponded with optical coherence tomography (OCT) findings of a rapidly progressing amoeboid-like lesion disrupting the ellipsoid zone and retinal pigment epithelium. Multimodal imaging, including fundus autofluorescence, OCT angiography, and indocyanine green angiography, was supportive of serpiginous choroiditis. After a comprehensive systemic workup, the diagnosis of macular serpiginous choroiditis was confirmed. No improvement was seen with high-dose steroids; therefore, an immunosuppressive regimen was initiated. **Conclusions:** An exaggerated choroidal inflammatory response may be triggered by a COVID-19 infection, although causation cannot be inferred. Retinal manifestations should be considered when assessing patients presenting with visual symptoms after COVID-19 infection.

## Introduction

Although respiratory disease from SARS-CoV-2 has been extensively documented, the ocular associations related to COVID-19 continue to be reported in the literature. Conjunctivitis remains the most frequent COVID-19 ocular manifestation (11.4%); however, an increasing number of cases of retinal and choroidal involvement have been reported.^1^ Diverse posterior segment manifestations have been found, including cotton-wool spots, retinal hemorrhages, posterior uveitis, retinal ischemia, and optic neuropathies.^2^ Some centers have had a significant increase in white-dot syndrome referrals during the pandemic, including serpiginous choroiditis.^3^

Serpiginous choroiditis is a rare, idiopathic, sight-threatening posterior uveitis often observed in healthy young or middle-aged individuals that tends to have a poor visual prognosis. It is characterized by asymmetric, recurrent, progressive inflammation of the choroid, resulting in atrophy of the choriocapillaris and retinal pigment epithelium (RPE).^4,5^ Although the exact pathogenesis is unclear, serpiginous choroiditis has been proposed to be immunogenic in nature, often preceded by a viral prodrome. Various infectious etiologies have been implicated, including tuberculosis, syphilis, and the herpes virus.^5^ COVID-19 has also been proposed as an immunologic trigger for reactivation of serpiginous choroiditis, with 1 documented case to date of recurrence after COVID-19 infection.^6^

We present what, to our knowledge, is the first case of macular serpiginous choroiditis onset after COVID-19 infection.

## Case Report

A 28-year-old, healthy man presented with severe vision loss in the left eye after 2 months of transient blurred vision lasting several hours in the left eye as well as binocular horizontal diplopia. Associated symptoms, including recurrent left-sided headaches and nausea, began during his deployment in the Navy, 1 month after polymerase chain reaction confirmed a COVID-19 infection. Eight months before the onset of symptoms, the patient noted a 4-day history of hand–foot–mouth disease. His medical history and ocular history were unremarkable, and he was not taking any medications. Of note, there was no history of ocular trauma or exposure to lasers or tuberculosis.

The patient initially presented to an ophthalmology clinic with a best-corrected visual acuity (BCVA) of 20/20 OD and 20/80 OS. Electroretinography was ordered to assess subtle parafoveal changes observed nasally in the left eye; however, his symptoms worsened before the test could be performed and he was referred to the retina service for a workup. On presentation to the retina clinic 1 month after symptom onset, the patient’s BCVA was 20/20 OD and counting fingers OS. The intraocular pressure was 18 mm Hg and 16 mm Hg, respectively. There was no relative afferent pupillary defect. There was grade 2 (Standardization of Uveitis Nomenclature criteria) anterior chamber inflammation in the left eye. Otherwise, an anterior segment examination was unremarkable. Fundoscopy showed significant atrophic pigmentary changes in the left macula. Of note, there was no vitritis or vasculitis. The fundoscopy examination of the right eye was normal.

**Figure 1.**
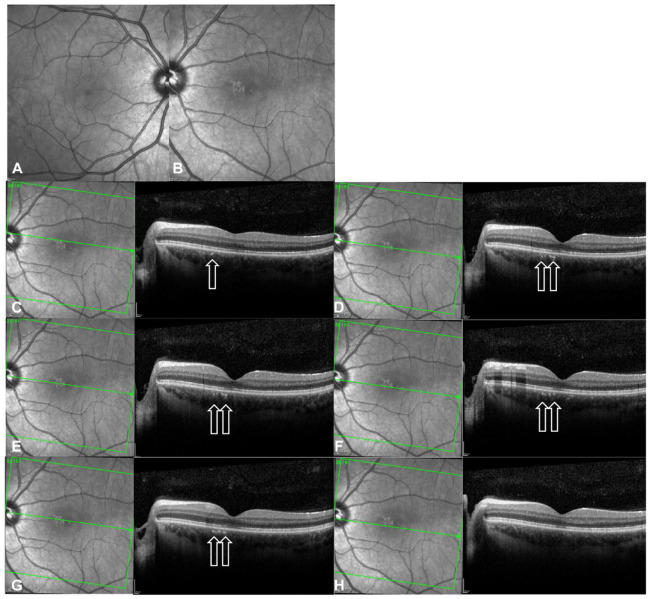
Near-infrared reflectance of (A) the right eye and (B) the left eye on initial presentation (1 month before referral to the retina clinic). (C–H) Near-infrared reflectance and optical coherence tomography (OCT) images show cuts of the left eye through the hyperreflective choroidal lesions. Arrows on OCT show the hyperreflective spots in the inner choroid that correspond with the hyperreflective areas nasal to the fovea seen on near-infrared reflectance. There is mild attenuation of the external limiting membrane, ellipsoid zone, and retinal pigment epithelium interdigitation lines. The neurosensory retina appears otherwise normal.

**Figure 2.**
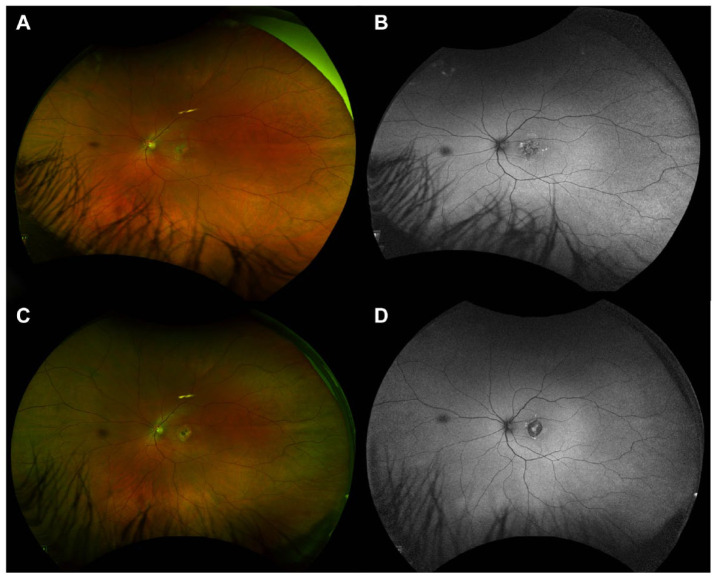
Serial progression of near-infrared reflectance and optical coherence tomography (OCT) images of the left eye performed at (A) the patient’s initial presentation to the eye clinic, (B) 4 weeks later, (C) 5 weeks later, and (D) 4 months after the initial visit. Near-infrared reflectance shows an irregular area of hyperreflectivity at the macula that corresponds with the hyperreflective foci in the outer retina and choroid on OCT. Over time, there is significant atrophy of the outer and inner retina with disruption of the ellipsoid zone and retinal pigment epithelium. Four months after the initial visit, a new pigment epithelial detachment is seen. In addition, there is a notable change in choroidal thickness over time as follows: 342 µm on the initial presentation, 378 µm at 1 month, and 233 µm at 4 months.

Spectral-domain optical coherence tomography (OCT) of the left macula at initial presentation showed mild attenuation of the foveal ellipsoid zone (EZ) (Figures 1 and 2A, open circle in inset). Focal hyperreflectivity of the inner choroid was noted nasal to the foveal center (Figure 2A, open arrows). Although hyperreflective foci was seen in the vitreous on OCT, no vitreous cells were seen clinically at the initial visit and subsequent visits. On near-infrared reflectance imaging, there was a group of multiple, small, round, hyperreflective lesions nasal to the fovea that localized to the hyperreflective choroidal findings on OCT (Figure 1). One month later, after observation only, the patient presented to the retina clinic with severe atrophy of the outer retina and inner retina that was apparent on OCT (Figure 2B). Near-infrared reflectance imaging showed expansion of the hyperreflective lesion through the macula, with an amoeboid-like shape (Figure 2B).

**Figure 3.**
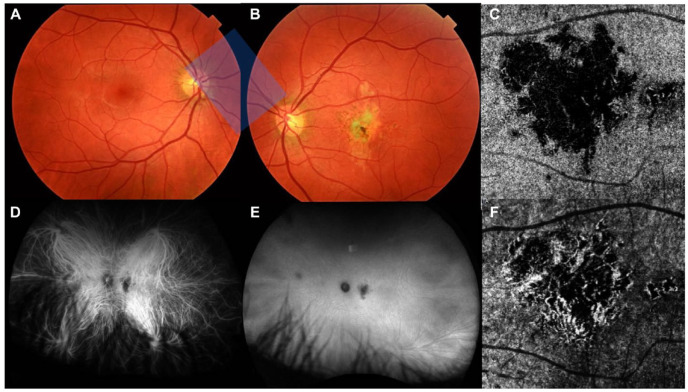
Multimodal imaging of the choroidal lesion 1 month after initial presentation. True color fundus photography of (A) the right eye and (B) the left eye shows a large unilateral gray–yellow macular lesion in the left eye. Indocyanine green angiography of the left eye shows hypofluorescence of the lesion during (D) the early phase and (E) the late phase of the study that corresponds with choroidal nonperfusion. Optical coherence tomography angiography en face analysis of (C) the choriocapillaris and (F) the choroid in the left eye shows severe ischemia of the affected choriocapillaris.

**Figure 4.**
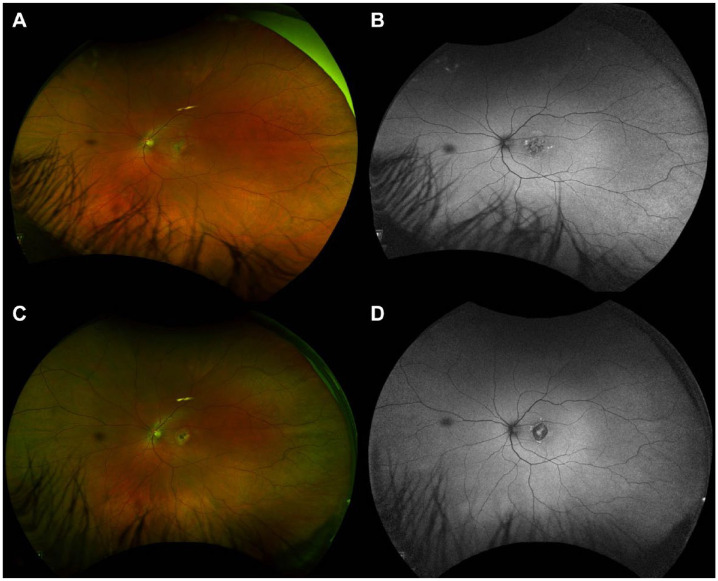
Widefield pseudocolour fundus photography and fundus autofluorescence of the left eye at 1 month (A and B) and 4 months (C and D) from initial presentation. Images show a large foveal hypoautofluorescent lesion surrounded by an irregular hyperautofluorescence border that evolved over time.

On fundus photography, the macular atrophic lesion appeared as a gray–yellow patch with indistinct borders (Figure 3, A and B). Fundus autofluorescence showed a corresponding hypoautofluorescent macular lesion surrounded by an amoeboid hyperautofluorescent ring projecting away from the fovea (Figure 4B). OCT angiography (OCTA) en face analysis showed severe inner choroidal ischemia at the lesion site (Figure 3, C and F). There was no macular neovascularization on OCTA. Indocyanine-green angiography (ICGA) analysis also showed early-phase and late-phase hypofluorescence of the lesion, indicating choroidal ischemia (Figure 3, D and E).

An extensive inflammatory and infectious workup, including a complete blood count, complement proteins C3/C4, C-reactive protein, serum angiotensin-converting enzyme, renal function tests, liver function tests, an antineutrophil antibody screen, and a vasculitis panel (antimyeloperoxidase, antiproteinase, antiglomerular basement membrane screens) yielded results within normal range or negative. Bartonella, toxocara serology, toxoplasma immunoglobulin (Ig) M and IgG, a Lyme disease antibody test, a syphilis enzyme immunoassay, an interferon gamma release assay tuberculosis test, and an HIV test were all negative. Aqueous fluid samples from the left eye were negative for adenovirus, cytomegalovirus, herpes simplex viruses 1 and 2 (HSV1, HSV2), and varicella zoster. Computed tomography of the head and magnetic resonance imaging of the brain were within normal limits.

Based on the clinical presentation, imaging, and laboratory testing, the patient was diagnosed with macular serpiginous choroiditis. He was started on 60 mg of oral prednisone daily. Because there was no subjective or objective improvement after 1 month and due to steroid-related side effects, he was tapered off the prednisone and started on 150 mg of oral azathioprine daily.

## Conclusions

Serpiginous choroiditis is a rare, idiopathic eye disease characterized by asymmetrically bilateral, recurrent, progressive inflammation of the choroid that results in atrophy of the choriocapillaris and RPE.^5^ To our knowledge, ours is the first documented case of macular serpiginous choroiditis presenting after a COVID-19 infection.

Macular serpiginous choroiditis was diagnosed after a comprehensive assessment to eliminate possible causes that may be masquerading as placoid maculopathy. The most common etiology, tuberculosis-related serpiginous-like choroiditis, was deemed to be unlikely because there was no known exposure to tuberculosis and the interferon gamma release assay bloodwork was negative. The differentiation between serpiginous choroiditis and serpiginous-like choroiditis is critical because the immunosuppressive drugs used for serpiginous choroiditis can reactivate and exacerbate tuberculosis infection. The observed macular presentation was distinct from that of tuberculosis-related serpiginous-like choroiditis, which typically involves stippled, multifocal lesions in the posterior pole and fundus periphery and is often associated with significant vitritis.^7^ Similarly, acute syphilitic posterior placoid chorioretinitis, which presents with large yellowish, multifocal placoid lesions and significant vitritis in immunocompromised hosts,^8^ was ruled out by laboratory work. In addition, the characteristic imaging hallmarks of acute syphilitic posterior placoid chorioretinitis were not seen on OCT, including reversible, irregular focal thickening and nodularity of the RPE with a disrupted EZ, and progressive hyperfluorescence with focal hypofluorescence or leopard spotting was not seen on fluorescein angiography.^8^ All other infectious workups were negative.

Ocular trauma was ruled out based on the patient’s history. The dendritic-like radiations of the lesion on the near-infrared reflectance and rapid enlargement of a central macular lesion without peripheral lesions raised the suspicion for handheld laser–induced maculopathy. However, the patient firmly denied any exposure to handheld or high-power lasers. In addition, there were no psychiatric comorbidities or clinical concern for intentional self-harm. There were also no atrophic laser burns in the iris. Despite EZ and RPE disruption, the OCT images lacked characteristic findings, such as the hyperreflective vertical streaks and angular signs of Henle fiber layer hyperreflectivity in the outer retina, which would be expected with choroidal ischemia and thermal injury to the layer.^9,10^

Inflammatory and placoid maculopathies were therefore at the top of the differential diagnoses. This was supported by the characteristic hypofluorescence on early-phase and late-phase ICGA (Figure 3, D and E) and choroidal flow voids on OCT-A (Figure 3, C and F). Unilateral acute idiopathic maculopathy was considered, especially in the context of the patient’s history of hand–foot–mouth disease. However, the prolonged 8-month onset after exposure makes this hypothesis less plausible.

Unilateral acute idiopathic maculopathy usually self-resolves spontaneously with near-complete recovery of vision, in contrast to the severe maculopathy observed in our patient (Figure 2, C and D). Also, multiple evanescent white-dot syndrome (MEWDS) and acute retinal pigment epitheliitis, known to have better clinical outcomes, were ruled out. Acute posterior multifocal placoid pigment epitheliopathy and serpiginous choroiditis thus emerged as the most likely diagnoses. Although they present similarly in the acute phase, acute posterior multifocal placoid pigment epitheliopathy is typically multifocal and symmetric and spontaneously resolves within weeks with mild to moderate residual RPE changes. However, the pronounced unilateral macular disruption observed on imaging were suggestive of serpiginous choroiditis. Ampiginous choroiditis, or relentless placoid chorioretinitis, was less likely because of its multifocal and widespread peripheral lesion distribution. We also determined that persistent placoid maculopathy was not consistent with our findings given the rapid decline in VA and the absence of bilateral disease.^11^ Therefore, after an extensive workup, the patient was diagnosed with macular serpiginous choroiditis.

Interestingly, the patient’s choroidal thickness changed over time; it was 342 µm on presentation, 378 µm 1 month later, and 233 µm 4 months later (Figure 2). Increasing choroidal thickness during acute inflammation and subsequent thinning after resolution is well-documented in posterior uveitis, consistent with an inflammatory process.^12^ The small pigment epithelial detachment observed on OCT (Figure 2C) was likely secondary to endovascular damage, increased vascular permeability, and blood–retinal barrier breakdown.^13^ Furthermore, the presence of choroidal hyperreflective foci (Figure 1) raises intriguing questions about the disease process. Although studies have shown similar findings in diabetic macular edema and dystrophies such as retina pigmentosa, there is no known evidence of similar patterns in patients with uveitis.^14,15^ It is speculated that these foci represent accumulations of activated microglial cells or migration of disrupted pigmented RPE cells.^14^

To our knowledge, there is only 1 other report of serpiginous choroiditis after COVID-19 infection, in which a 41-year-old woman presented with multifocal peripapillary lesions in the left eye consistent with disease reactivation 1 month after a mild infection.^6^ Although most serpiginous choroiditis cases (80%) manifest with peripapillary lesions extending outward from the optic disc, our patient had a new onset of the rare macular variant.^5^ This variant is marked by an unfavorable prognosis attributed to early foveal involvement. A growing body of research proposes that SARS-CoV-2 may serve as a proinflammatory trigger in susceptible hosts, inducing autoimmune and autoinflammatory dysregulation.^6,16,17^ The choroid’s rich vascular supply naturally fosters a favorable environment for inflammatory processes, but the exact mechanisms remain an active area of research.

Various forms of posterior uveitis have been reported after COVID-19 infection. To date, there have been 2 reported instances of ampiginous choroiditis, an entity sharing features of serpiginous choroiditis. The patients, both in their early 20s, developed bilateral ampiginous choroiditis approximately 1 week after COVID-19 infection.^17,18^ Similar to the report from Providência et al,^6^ 1 patient had preexisting retinal lesions, suggesting that COVID-19 may have triggered a reactivation. Our patient did not have evidence of previously known retinal disease. Furthermore, there have been reports linking COVID-19 to other forms of inflammatory choroidopathies, including acute posterior multifocal placoid pigment epitheliopathy, punctate inner choroidopathy, and multifocal choroiditis.^16,19,20^ In addition, numerous patients have presented with MEWDS after COVID-19 infection.^21–24^ Although a subset of patients had evidence of previous inflammatory ocular disease, many were young healthy patients presenting for the first time. Some presented acutely during or immediately after COVID-19 infection, while others, like our patient, experienced a delayed onset of weeks to months.

The temporal association between our patient’s COVID-19 infection and the onset of serpiginous choroiditis raises intriguing questions about the role of the virus in triggering or exacerbating such conditions. Causation cannot be definitively established; however, our findings echo the sentiments of other researchers who have proposed a potential link between COVID-19 and choroidal inflammation. Many studies have noted optic nerve and vascular changes occurring weeks to months after COVID-19 infection, including acute posterior multifocal placoid pigment epitheliopathy starting 6 weeks after infection and MEWDS symptoms developing 10 weeks after infection.^22,25,26^ However, these timelines are not exhaustive because retinopathies may initially go undetected when asymptomatic. Our observations align with this concept, showing that chorioretinal changes can manifest subacutely as the disease progresses. Nonetheless, serpiginous choroiditis is extremely rare, comprising 1% to 5% of all uveitis cases in areas of nonendemic tuberculosis, while COVID-19 is an increasingly common disease.^27^

The initial treatment for active serpiginous choroiditis lesions involves systemic corticosteroid therapy, while immunosuppressive therapy is crucial for preventing recurrences. Our patient was started on a daily oral regimen of azathioprine 150 mg to reduce the risk for recurrence and potential damage to the right eye. Unfortunately, the visual prognosis is usually quite poor, particularly in cases characterized by extensive atrophy and scarring.

In conclusion, we present what to our knowledge is the first case of macular serpiginous choroiditis with an onset after a COVID-19 infection. Our findings align with the existing literature on ocular complications after COVID-19 infection; however, the atypical macular variant in this case makes it unique. Further research is required to elucidate the role of COVID-19 infection in the development of this rare disease.

## References

[1] Binotti W Hamrah P. COVID-19-related conjunctivitis review: clinical features and management. Ocul Immunol Inflamm. 2023;31(4):778-784. doi:10.1080/09273948.2022.2054432 35394858

[2] Sen S Kannan NB Kumar J , et al. Retinal manifestations in patients with SARS-CoV-2 infection and pathogenetic implications: a systematic review. Int Ophthalmol. 2022;42(1):323-336. doi:10.1007/s10792-021-01996-7 34379290 PMC8356207

[3] Jones NP Pockar S Steeples LR. Changing trends in uveitis in the United Kingdom: 5000 consecutive referrals to a tertiary referral centre. Ocul Immunol Inflamm. 2023;31(5):921-926. doi:10.1080/09273948.2022.2067067 35442852

[4] Dutta Majumder P Biswas J Gupta A . Enigma of serpiginous choroiditis. Indian J Ophthalmol. 2019;67(3):325-333. doi:10.4103/ijo.IJO_822_18 30777946 PMC6407399

[5] Lim WK Buggage RR Nussenblatt RB. Serpiginous choroiditis. Surv Ophthalmol. 2005;50(3):231-244. doi:10.1016/j.survophthal.2005.02.010 15850812

[6] Providência J Fonseca C Henriques F Proença R. Serpiginous choroiditis presenting after SARS-CoV-2 infection: a new immunological trigger? Eur J Ophthalmol. 2022;32(1):NP97-NP101. doi:10.1177/1120672120977817 33267645

[7] Vasconcelos-Santos DV Rao PK Davies JB Sohn EH Rao NA. Clinical features of tuberculous serpiginouslike choroiditis in contrast to classic serpiginous choroiditis. Arch Ophthalmol. 2010;128(7):853-858. doi:10.1001/archophthalmol.2010.116 20625045

[8] Neri P Pichi F. Acute syphilitic posterior placoid chorioretinitis: when the great mimicker cannot pretend any more; new insight of an old acquaintance. J Ophthalmic Inflamm Infect. 2022;12(1):9. doi:10.1186/s12348-022-00286-2 35192047 PMC8864036

[9] Bhavsar KV Michel Z Greenwald M Cunningham ET Freund KB. Retinal injury from handheld lasers: a review. Surv Ophthalmol. 2021;66(2):231-260. doi:10.1016/j.survophthal.2020.06.006 32628946

[10] Ramtohul P Cabral D Sadda S Freund KB Sarraf D. The OCT angular sign of Henle fiber layer (HFL) hyperreflectivity (ASHH) and the pathoanatomy of the HFL in macular disease. Prog Retin Eye Res. 2023;95:101135. doi:10.1016/j.preteyeres.2022.101135 36333227

[11] Golchet PR Jampol LM Wilson D Yannuzzi LA Ober M Stroh E. Persistent placoid maculopathy: a new clinical entity. Ophthalmology. 2007;114(8):1530-1540. doi:10.1016/j.ophtha.2006.10.050 17678692

[12] Baltmr A Lightman S Tomkins-Netzer O. Examining the choroid in ocular inflammation: a focus on enhanced depth imaging. J Ophthalmol. 2014;2014:459136. doi:10.1155/2014/459136 25024846 PMC4082870

[13] Green K Paterson CA Cheeks L Slagle T Jay WM Aziz MZ. Ocular blood flow and vascular permeability in endotoxin-induced inflammation. Ophthalmic Res. 1990;22(5):287-294. doi:10.1159/000267037 2090983

[14] Nagasaka Y Ito Y Ueno S Terasaki H. Number of hyperreflective foci in the outer retina correlates with inflammation and photoreceptor degeneration in retinitis pigmentosa. Ophthalmol Retina. 2018;2(7):726-734. doi:10.1016/j.oret.2017.07.020 31047383

[15] Roy R Saurabh K Shah D Chowdhury M Goel S. Choroidal hyperreflective foci: a novel spectral domain optical coherence tomography biomarker in eyes with diabetic macular edema. Asia Pac J Ophthalmol (Phila). 2019;8(4):314-318. doi:10.1097/APO.0000000000000249 31397675 PMC6727920

[16] Nicolai M Carpenè MJ Lassandro NV , et al. Punctate inner choroidopathy reactivation following COVID-19: a case report. Eur J Ophthalmol. 2022;32(4):NP6-NP10. doi:10.1177/11206721211028750 PMC9294619 34219492

[17] Carvalho EM Teixeira FHF de Carvalho Mendes Paiva A Santos NS Biancardi AL Curi ALL . Bilateral ampiginous choroiditis following confirmed SARS-CoV-2 infection. Ocul Immunol Inflamm. 2023;31(4):843-846. doi:10.1080/09273948.2022.2049317 35404726

[18] Tom ES McKay KM Saraf SS. Bilateral ampiginous choroiditis following presumed SARS-CoV-2 infection. Case Rep Ophthalmol Med. 2021;2021:1646364. doi:10.1155/2021/1646364 34367705 PMC8346294

[19] Fischer NA Wann RC Crosson JN. Acute posterior multifocal placoid pigment epitheliopathy following COVID-19 infection. Am J Ophthalmol Case Rep. 2023;29:101790. doi:10.1016/j.ajoc.2022.101790 36597447 PMC9800013

[20] de Souza EC de Campos VE Duker JS. Atypical unilateral multifocal choroiditis in a COVID-19 positive patient. Am J Ophthalmol Case Rep. 2021;22:101034. doi:10.1016/j.ajoc.2021.101034 33623832 PMC7893242

[21] Gallo B Talks JS Pandit RJ Browning AC. Multiple evanescent white dot syndrome and choroidal neovascularization following SARS-COV-2 infection in a patient on dabrafenib and trametinib. Ocul Immunol Inflamm. 2023;31(3):641-648. doi:10.1080/09273948.2022.2042320 35226581

[22] Jain A Shilpa IN Biswas J. Multiple evanescent white dot syndrome following SARS-CoV-2 infection - a case report. Indian J Ophthalmol. 2022;70(4):1418-1420. doi:10.4103/ijo.IJO_3093_21 35326071 PMC9240486

[23] Smeller L Toth-Molnar E Sohar N. White dot syndrome report in a SARS-CoV-2 patient. Case Rep Ophthalmol. 2022;13(3):744-750. doi:10.1159/000526090 36845457 PMC9944209

[24] Adzic Zecevic A Vukovic D Djurovic M Lutovac Z Zecevic K . Multiple evanescent white dot syndrome associated with coronavirus infection: a case report. Iran J Med Sci. 2023;48(1):98-101. doi:10.30476/IJMS.2022.95007.2632 36688189 PMC9843462

[25] Olguín-Manríquez F Cernichiaro-Espinosa L Olguín-Manríquez A Manríquez-Arias R Flores-Villalobos EO Kawakami-Campos PA. Unilateral acute posterior multifocal placoid pigment epitheliopathy in a convalescent COVID-19 patient. Int J Retina Vitreous. 2021;7(1):41. doi:10.1186/s40942-021-00312-w 34034832 PMC8148402

[26] Vélez Cevallos MA Vásquez AM . Alterations in the optic nerve and retina in patients with COVID-19. A theoretical review. Arch Soc Esp Oftalmol (Engl Ed). 2023;98(8):454-469. doi:10.1016/j.oftale.2023.06.015 37369321 PMC10290763

[27] Nazari Khanamiri H Rao NA . Serpiginous choroiditis and infectious multifocal serpiginoid choroiditis. Surv Ophthalmol. 2013;58(3):203-232. doi:10.1016/j.survophthal.2012.08.008 23541041 PMC3631461

